# Obstetric outcomes of women vaccinated with the COVID-19 vaccine (≥1 dose): A single-center retrospective cohort study of pregnant Chinese women

**DOI:** 10.1097/MD.0000000000039053

**Published:** 2024-07-26

**Authors:** Mei Zhang, Shuyu Wu, Dejing Wang

**Affiliations:** a Department of Reproduction Medicine, Affiliated Hospital of Zunyi Medical University, Zunyi, Guizhou Province, China; b Department of Obstetrics and Gynecology, Affiliated Hospital of Zunyi Medical University, Zunyi, Guizhou Province, China.

**Keywords:** COVID-19, obstetric outcomes, pregnancy, vaccine

## Abstract

In the context of the coronavirus disease 2019 (COVID-19) pandemic, the quickly developed COVID-19 vaccine may cause various adverse reactions, especially in special groups, such as pregnant women. However, many pregnant women have concerns regarding vaccination in terms of safety for themselves and their neonates. Therefore, we studied the obstetric outcomes of pregnant women in Zunyi, China. In this retrospective study, we examined differences between pregnant women who were vaccinated and pregnant women who were not vaccinated/vaccinated at the end of pregnancy. In addition, we collected and retrieved the literature related to the COVID-19 vaccine and pregnancy outcomes from PubMed. Among concluded women, 369 were included in the study group and 231 were included in the control group. There were no differences in the baseline characteristics, labor rate, or rates of poor pregnancy outcomes between the 2 groups. Based on the adverse reaction and obstetric outcome data of pregnant women who received the COVID-19 vaccine in China, the vaccine does not raise any safety concerns. This result is the same as that of other countries we summarized. The COVID-19 vaccine has no effect on pregnancy outcomes.

## 1. Introduction

Coronavirus disease 2019 (COVID-19), characterized as a disease caused by a virus with constant variation, has led to severe global morbidity and mortality.^[1,2]^ With the hard work of vaccine researchers, good control has been achieved in China.^[3]^

The COVID-19 adenovirus vaccine, produced by CanSino Biologics Biological Co. Ltd (CanSino Biologics, Tianjin, China), is the only adenovirus vaccine that has been conditionally approved for marketing in China. Research has shown that a single vaccine dose can cause significant immune responses in adults aged 18 years and above, including humoral and cellular immunity.^[4]^

An inactivated vaccine contains a virus that has been inactivated by physical or chemical methods; in these vaccines, the immunogenicity is retained, but the infectivity is not. In a phase I/II clinical trial, subjects who received the Kexing vaccine were divided into 2 age groups: those aged 18 to 59 years and those aged ≥60 years. According to the research results, the vaccine showed good safety and immunogenicity for subjects in both age groups.^[5,6]^ *The Lancet* published the interim analysis results of the Kexing vaccine phase III clinical trial conducted in Türkiye online. The trial included 10,000 volunteers aged 18 to 59 years. The results showed that 14 days after inoculation with 2 doses of the vaccine, the effective rate for COVID-19 prevention was 83.5%, and the effective rate for the prevention of COVID-19-related hospitalization was 100%.^[7]^

The recombinant protein vaccine integrates the receptor binding region of the coronavirus S protein into Chinese hamster ovary cells through genetic recombination and expresses the receptor binding region dimer in vitro; an aluminum hydroxide adjuvant is added to improve immunogenicity.^[8]^

However, many special groups, especially pregnant women, are not sure whether vaccines made in China are safe.^[9]^ The International Federation of Gynecology and Obstetrics reports that many women of childbearing age are hesitant to receive the vaccine.^[10]^ Fertility safety data on unintended pregnancies during the COVID-19 pandemic are needed. Data results for a growing number of pregnant women who are willing to be vaccinated can help women of childbearing age make informed decisions.^[11]^ Therefore, to collect the relevant research data and draw corresponding conclusions, clinical trials of women who are willing to receive the COVID-19 vaccine are crucial.^[12]^ More clinical studies are needed to verify the safety of these vaccines. This retrospective cohort study was conducted in the Affiliated Hospital of Zunyi Medical University in Guizhou Province, which is one of the major tertiary hospitals in the province. Cases were collected from the hospital system by the keywords “early pregnancy” and “pregnancy examination.” We thus analyzed obstetric outcomes and adverse reactions after vaccination to provide a clinical basis for the safety of the COVID-19 vaccine.

## 2. Methods

### 2.1. Design

A retrospective analysis of pregnant women (1091 patients) who presented to the Affiliated Hospital of Zunyi Medical University between January 1, 2020, and September 9, 2022, was conducted through pregnancy-related telephone visits to collect data on vaccination and pregnancy outcomes. According to the time of COVID-19 vaccination and pregnancy, the women were divided into 2 groups: the study group, who were vaccinated before or during pregnancy (369 patients), and the control group, who were vaccinated after pregnancy or never vaccinated (231 patients). Women with a history of COVID-19 infection, an unknown vaccination status, missing follow-up information on pregnancy outcomes, and medication use were excluded. Through telephone follow-up, participants were asked about their side effects, including muscular soreness, localized pain in the injection area, uterine contractions, severe exhaustion, erythema, and swelling. The questionnaires were completed by telephone, and the relevant data were sorted and analyzed. Since this was an observational study, the Zunyi Medical University Research Ethics Committee confirmed that no ethical approval was needed. When we collected participant information, we obtained verbal consent. Finally, we will summarize and analyze the literature on the impact of the COVID-19 vaccine on pregnancy outcomes collected from PubMed.

### 2.2. Data analysis

Statistical analysis was performed using SPSS 29.0 (IBM Corp., Armonk, Version 29.0). Normally distributed measurements are expressed as the mean ± standard deviation ($\bar{x}\pm s$). For univariate analyses, an independent-sample *t* test was used for normally distributed continuous variables, and the Mann–Whitney *U* test was used for non-normally distributed continuous variables. Categorical variables are described as percentages, and the chi-square test or Fisher exact test was performed. To evaluate the correlation between poor obstetric outcomes (including abortion, ectopic pregnancy, and biochemistry) and adverse reactions, rank sum tests were used for nonlinear variables, and linear tests were used for linear variables with a non-normal distribution. Adjusted odds ratios (aORs) and 95% confidence intervals (CIs) were used for poor obstetric outcomes.

## 3. Results

### 3.1. Descriptive analysis and imputation

**Figure 1.**
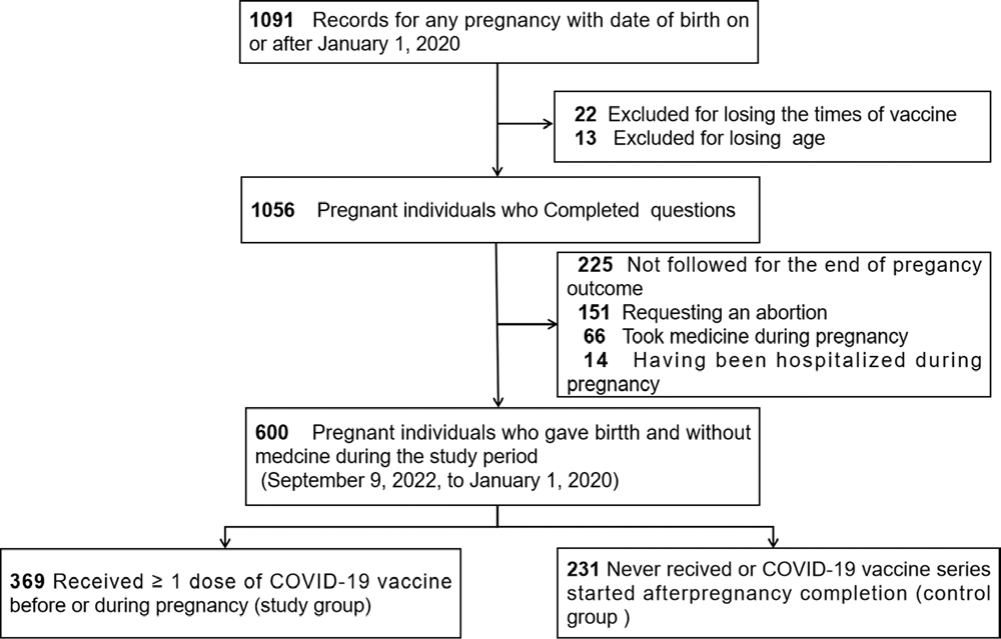
Flowchart showing the enrollment of the pregnant women involved in our study. COVID-19 = coronavirus disease 2019.

Of the 1091 pregnant women who presented to our hospital for examination, 35 with incomplete questionnaires, 305 with incomplete follow-up information on pregnancy outcomes and medication use during pregnancy, and 151 who requested an abortion were excluded. Overall, we included 600 of the 1091 pregnant women in the study (Fig. 1). Most pregnant women were under 35 years old (86.33%) and were vaccinated with the Vero Cell COVID-19 vaccine (64.66%). The incidence rate of poor pregnancy outcomes (including abortion, ectopic pregnancy, and biochemistry) in our hospital is 10.50% (Table 1). The adverse reaction rate was 7.00%, and the live birth rate was 38.17%. For most patients, adverse reactions, complications, and a history of poor pregnancy were not present. Although there are more people in the control group receiving the Vero Cell COVID-19 vaccine (*P* = .008), this may be influenced by the vaccine development process and market circulation. Overall, there was no significant difference in the proportion of these data between the 2 groups (*P* > .05).

**Table 1:** Baseline characteristics of the women in the study group and control group.

	Overall	Study group	Control group	*P*
No. of cases	600	369	231	—
Age (yr)				
≤35	518 (86.33%)	322 (87.26%)	196 (84.85%)	0.402
35–40	54 (9.00%)	29 (7.86%)	25 (10.82%)	0.217
>40	28 (4.67%)	18 (4.88%)	10 (4.33%)	0.756
Vaccine type				
Adenovirus type 5 vector	32 (6.02%)	17 (5.26%)	15 (7.18%)	0.317
Vero Cell	344 (64.66%)	196 (60.68%)	148 (70.81%)	0.008
CHO Cell	156 (29.32%)	110 (34.06%)	46 (22.01%)	0.07
Poor pregnancy history				
No	537 (89.50%)	335 (90.79%)	202 (87.45%)	0.194
Yes	63 (10.50%)	34 (9.21%)	29 (12.55%)	—
Adverse reaction				
No	558 (93.00%)	349 (94.58%)	209 (90.48%)	0.055
Yes	42 (7.00%)	20 (5.42%)	22 (9.52%)	—
Obstetric outcome				
Poor outcome	229 (38.17%)	145 (39.30%)	84 (36.36%)	0.472
Labor	371 (61.83%)	224 (60.70%)	147 (63.63%)	—
Complication				
No	522 (87.00%)	328 (88.89%)	194 (83.98%)	0.082
Yes	78 (13.00%)	41 (11.11%)	37 (16.02%)	—

CHO = Chinese hamster ovary.

### 3.2. Chi-square test

The age of the study group was younger than that of the control group (29.72 ± 5.30 vs 30.09 ± 5.26 years), but the difference was not statistically significant (*P* > .05) (Table 2). The 2 groups accounted for the largest proportion of labor, followed by abortion (including spontaneous and induced abortion), ectopic pregnancies, and biochemical pregnancies (the lowest proportion). The top 3 adverse reactions were muscle soreness, localized pain in the injection area, and swelling.

**Table 2:** Chi-square test of the main variables between the 2 groups.

Factors	Study group (N = 369)	Control group (N = 231)	*P*
Age	29.72 ± 5.30	30.09 ± 5.26	0.409
Adverse reaction			
Muscular soreness	10 (2.71%)	8 (3.46%)	0.779
Local pain	8 (2.17%)	9 (3.90%)	0.214
Uterine contraction	2 (0.54%)	0	0.694
Severe exhaustion	2 (0.54%)	1 (0.43%)	1
Erythra	2 (0.54%)	1 (0.43%)	1
Swelling	2 (0.54%)	2 (0.87%)	1
Obstetric outcome			
Labor	224 (60.70%)	147 (63.64%)	0.472
Abortion	133 (36.04%)	76 (32.90%)	0.432
Biochemical pregnancy	5 (1.56%)	4 (1.73%)	0.981
Ectopic pregnancy	7 (1.90%)	4 (1.73%)	1

### 3.3. Regression analysis

**Figure 2.**
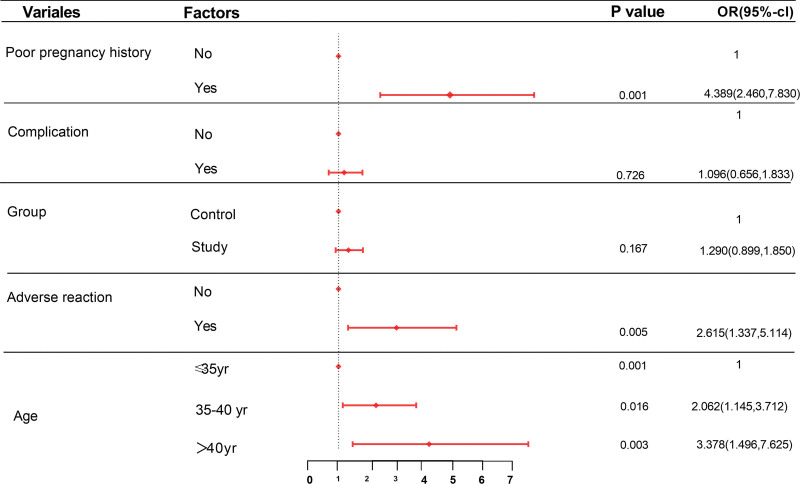
Adjusted odds ratios (ORs) and 95% confidence intervals (CIs) for poor obstetric outcomes. The regression model included all variables listed.

Regression analysis based on a pregnancy loss outcome was performed. According to the regression analysis, a history of poor pregnancy outcomes, adverse reactions, and advanced age seem to be the most dangerous factors associated with abortion. The risk factor for a history of poor pregnancy outcomes showed the highest aOR (aOR: 4.389, 95% CI: 2.460–7.830), while there was no difference for complications and groups (aOR: 1.096, 95% CI: 0.656–1.833; aOR: 1.290, 95% CI: 0.899–1.850). Adverse reactions showed a statistically significant effect on the abortion rate (aOR: 2.615, 95% CI: 1.337–5.114). The predicted abortion rate by age, ranging from 35 to 40 years (95% CI: 2.062–3.712) to older than 40 years (95% CI: 1.496–7.625), showed that advanced age was the second most relevant risk factor. In addition, vaccination status was not a significant factor in our study (Fig. 2).

### 3.4. Article review

We collected 10 clinical research articles in PubMed that accurately matched COVID-19 vaccines and pregnancy outcomes (Table 3).

**Table 3:** Features of studies included in the review.

References	Period considered	Sample size	Study design	Inclusion criteria	Vaccine type	Location	Obstetric outcomes
Shimabukuro et al^[11]^	December 2020–February 2021	827 (827 vs 0)	Retrospective	All women aged 16–54 yr of age identified as pregnant	19252 Pfizer‐BioNTech, 16439 Moderna	US	*,‡
Dick et al^[13]^	December 2020–July 2021	2358 (2305 vs 3313)	Retrospective	All known pregnant women	Pfizer-BioNTech	Israel	‡,§
Wainstock et al^[14]^	January 2021–June 2021	4399 (913 vs 3486)	Retrospective	Women who delivered singletons	Pfizer-BioNTech	Israel	‡
Theiler et al^[15]^	December 2020–April 2021	2002 (140 vs 1862)	Prospective	Women aged 16–55 yr with a delivery event	1 Janssen 12 Moderna 27 Pfizer-BioNTech	US	‡
Bookstein Peretz et al^[1]^	January–February 2021	650 (390 vs 260)	Retrospective	Women with singleton deliveries	Pfizer-BioNTech	Israel	‡,§
Lipkind et al^[16]^	December 2020–July 2021	46,079 (10,064 vs 36,015)	Retrospective	Women with singleton deliveries	Pfizer- BioNTech	US	§
Rottenstreich et al^[17]^	January 2021–April 2021	1775 (712 vs 1063)	Retrospective	Women who gave birth at >24 wk of gestation	Pfizer‐BioNTech	Israel	†
Blakeway et al^[18]^	March 2021–July 2021	1328 (140 vs 1188)	Retrospective	Women who gave birth at St George’s University Hospitals National Health Service Foundation Trust	127 (Moderna, Pfizer-BioNTech) 13 Oxford-AstraZeneca	UK	†
Sadarangani et al^[19]^	Nov 2021–December 2021	8705 (5597 vs 3108)	Prospective	Pregnant females aged 15–49 yr	3414 Pfizer-BioNTech; 2183 mRNA-1273	Canada	‡
Kharbanda et al^[20]^	November 2021–June 2022	285,079 (14,226 vs 270,853)	Retrospective	Women aged 16–49 yr	Moderna, Pfizer-BioNTech	US	†

COVID-19 = coronavirus disease 2019.

* Compared with the normal miscarriage rate, the miscarriage rate did not increase after vaccination.

† Compared with the normal group, the abortion rate did not increase after vaccination.

‡ Compared with the control group, there was no significant change in the occurrence of adverse events after vaccination.

§ COVID-19 vaccination is not associated with premature birth.

These 10 studies included 130,994 pregnant women, 67,966 (51.91%) of whom received the COVID-19 vaccine, while 63,082 (48.1%) pregnant women did not receive it. The majority of studies were performed in the US^[11, 15, 16, 20]^ and Israel^[1, 13, 14, 17]^; one study was performed in the UK^[18]^ and another in Canada.^[19]^ Only one study was prospective,^[19]^ whereas the others were retrospective observational analyses. Only one study that evaluated cohorts of women, including vaccines other than mRNA-based, described 1 woman vaccinated with a Janssen (Ad.26.COV2. S) COVID-19 vaccine.^[15]^ The results of all the above studies showed that the COVID-19 vaccine will not affect the pregnancy outcome.

## 4. Discussion

The results of several clinical studies have demonstrated the safety of the COVID-19 vaccine and its effectiveness in preventing COVID-19 infection to some extent. Vaccination during pregnancy may affect maternal and infant health.^[6, 21, 22]^ In this retrospective cohort study, vaccination with 3 kinds of vaccines during the prenatal period was not associated with immediate adverse pregnancy outcomes.

Based on a vaccine trial of pregnant women vaccinated with the new COVID-19 vaccine that analyzed maternal health and fetal and newborn development, there is almost no reliable evidence that vaccination during pregnancy affects pregnancy outcomes.^[23]^ This belief hinders health care professionals from providing evidence-based advice on the safety and effectiveness of vaccination to these populations.^[24]^ However, most pregnancies are unexpected, and women may not be aware of early pregnancy before vaccination against the novel coronavirus. Therefore, vaccination information during pregnancy is very important to monitor the safety and effectiveness of the vaccine.^[25]^ Foreign studies have found that COVID-19 vaccination in the third trimester is not associated with adverse maternal outcomes and that it reduces the risk of adverse neonatal outcomes.^[11, 17]^ For mRNA vaccines, the antibody response rate is almost 100%.^[26, 27]^ Sufficient evidence has shown that the vaccine does not cause life-threatening side effects in pregnant women or fetuses. If vaccination occurs during pregnancy, antibodies may cross the placenta and may passively immunize the fetus. The vaccination of women in the first trimester of pregnancy may produce greater infant immunity.^[28]^ Combined with the actual situation in China, Chinese experts suggest that pregnancy is not a contraindication to the COVID-19 vaccine (especially the inactivated vaccine). In the case of the risk of COVID-19 infection, COVID-19 vaccination is recommended according to the procedure, including in early, middle, and late pregnancy. Pregnant or lactating women can also be vaccinated with the COVID-19 vaccine according to routine protocols and can normally feed their infants after vaccination.^[29]^ Women of childbearing age who are planning pregnancy or are pregnant can rest assured that multiple studies suggest that vaccination does not impair fertility.^[30]^ In our single-center retrospective cohort study, vaccination with the COVID-19 vaccine did not seem to be a risk factor for adverse pregnancy outcomes. Instead, advanced age increased the risk of poor obstetric outcomes by >3.378 times, and the risk seemed to increase with increasing age. A history of poor pregnancy outcomes increased the risk by 4.389 times. Therefore, COVID-19 vaccination is safe for pregnant women, but the relationship between adverse reactions and poor obstetric outcomes still needs further study. This study has certain limitations. This study was conducted in a single center, the Affiliated Hospital of Zunyi Medical University. Therefore, clinical trials with large samples are needed to evaluate the impact of vaccination produced in China on pregnancy outcomes.

## 5. Conclusions

The COVID-19 vaccine has now been inoculated by most people in China in recent years. Research is enhancing our understanding of whether these vaccines have adverse effects on pregnant women. Our research supports the view that the COVID-19 vaccine is safe for pregnant women.

## Author contributions

**Formal analysis:** Mei Zhang, Shuyu Wu.

**Methodology:** Mei Zhang.

**Visualization:** Mei Zhang.

**Writing—original draft:** Mei Zhang.

**Conceptualization:** Shuyu Wu.

**Funding acquisition:** Dejing Wang.

**Writing—review and editing:** Dejing Wang.
